# The dual role of short fatty acid chains in the pathogenesis of autoimmune disease models

**DOI:** 10.1371/journal.pone.0173032

**Published:** 2017-02-24

**Authors:** Miho Mizuno, Daisuke Noto, Naoko Kaga, Asako Chiba, Sachiko Miyake

**Affiliations:** 1 Department of Immunology, Juntendo University School of Medicine, Bunkyo-ku, Tokyo, Japan; 2 Laboratory of Proteomics and Biomolecular Science Research Support Center, Juntendo University Graduate School of Medicine, Bunkyo-ku, Tokyo, Japan; Wayne State University, UNITED STATES

## Abstract

Autoimmune diseases are influenced by both genetic and environmental factors. The gut environment has attracted much attention as an essential component that modulates immune responses, and therefore immune-mediated disorders, such as autoimmune diseases. Growing evidence suggests that microbiota and their metabolites are critical factors for immune modulation. Recently, we reported that the microbiome in patients with multiple sclerosis, an autoimmune disease targeting the myelin sheath of the central nervous system, is characterized by a reduction of bacteria belonging to *Clostridia* clusters IV and XIVa, which are potent producers of short-chain fatty acids (SCFAs) by fermentation of indigestible carbohydrates. In the present study, we investigated the role of SCFAs in the regulation of inflammation. We demonstrated that oral administration of SCFAs ameliorated the disease severity of systemic autoimmune inflammatory conditions mediated by lymphocytes such as experimental autoimmune encephalitis and collagen-induced arthritis. Amelioration of disease was associated with a reduction of Th1 cells and an increase in regulatory T cells. In contrast, SCFAs contributed to the exaggeration of K/BxN serum transfer arthritis, representing the effector phase of inflammation in rheumatoid arthritis. An increased understanding of the effect of microbiota metabolites will lead to the effective treatment and prevention of systemic inflammatory disorders.

## Introduction

Extensive studies have revealed the involvement of both genetic and environmental factors in the pathogenesis of autoimmune diseases, although the precise mechanisms remain unclear. The gut environment is an important factor for the development and modulation of the immune system. Notably, the relationship between gut microbiota and systemic immune responses has attracted much attention with regard to the pathogenesis of immune-mediated disorders including autoimmune diseases [1]. Experimental approaches using germ free conditions or antibiotic treatment have proven that alteration of the gut microbiota is a potential risk factor for developing autoimmune diseases [2–4]. More recently, certain species of bacteria, such as segmented filamentous bacteria, have been shown to contribute to the augmentation of autoimmune diseases associated with the induction of Th17 cells [4–6]. In addition to disease-promoting bacteria, gut bacteria that potentially suppress inflammation have also been reported. Human feces-derived *Clostridia* clusters XIVa and IV, as well as *Bacteroides fragilis* have been shown to suppress inflammatory conditions through the induction of Foxp3^+^ regulatory T cells (Tregs) [7–9].

These experiments raised the possibility that an altered gut microbiota is an environmental risk factor for autoimmune diseases. We recently reported the presence of dysbiosis in the gut microbiota of patients with multiple sclerosis (MS), an autoimmune disease affecting the central nervous system [10]. The dysbiosis of gut microbiota in MS patients was characterized by a reduction of species belonging to *Clostridia* XIVa and IV clusters. These species produce short chain fatty acids (SCFAs) including acetate, propionate, and butyrate by the fermentation of soluble fiber contained in the diet. Recent studies revealed that the administration of SCFAs into rodent inflammatory models inhibited the disease by increasing the number of Tregs [11–15].

To investigate the effect of SCFAs in autoimmune disease models, we administrated SCFAs into mice and induced experimental autoimmune encephalomyelitis (EAE), type II collagen-induced arthritis (CIA) or antibody-induced arthritis (AIA). SCFAs suppressed EAE in association with an increase in Tregs and a decrease in Th1 cells. In contrast, SCFAs augmented the disease severity of AIA induced by the transfer of serum obtained from KRN TCR-transgenic mice crossed with NOD (K/BxN) mice. These results suggested that the oral administration of SCFAs inhibited systemic autoimmune diseases such as EAE and CIA. However, SCFAs may enhance the effector phase of inflammation.

## Material and methods

### Mice

C57BL/6 (B6) mice were purchased from CLEA Laboratory Animal Corp (Tokyo, Japan). DBA/1J mice were purchased from the Oriental Yeast Company. KRN TCR-transgenic mice were kindly provided by Drs. Christophe Benoist and Diane Mathis (Harvard Medical School, Boston, MA). This study was approved by the Animal Experimental Committee of the Juntendo University Graduate School of Medicine (Permit Number: 280042 and 280066). Mice were maintained in specific pathogen-free conditions in accordance with institutional guidelines. All mice were sacrificed by decapitation under isoflurane anesthesia.

### Diet and SCFA treatment

Mice were fed either normal chow containing 5% cellulose (Clea diet AIN-93G) or a low-fiber diet (Clea diet AIN-93G without cellulose). When studying the effect of a high-fiber diet, mice were given a low-fiber diet supplemented with 30% pectin (Wako, Tokyo, Japan). All diets were purchased from CLEA Laboratory Animal Corp. Mice were adapted to low-fiber or high-fiber chow 2 weeks before immunization and throughout the study.

When studying the effect of SCFAs, mice were given sodium acetate or sodium propionate or sodium butyrate (Wako) in the drinking water at a final concentration of 200 mM for 3 weeks before immunization and throughout the study. Mice were given normal drinking water in the control group.

### Cecal SCFA quantification

SCFAs were extracted based on the procedure reported by Trompette et al [14]. Briefly, 100 mg of cecum or feces were homogenized in 400 μl of H_2_O containing hexanoic acid (methyl-d_3_, Cambridge Isotope Laboratories, Tewksbury, MA) used as an internal standard. Then, 80 μl of 25% meta-phosphoric acid (Sigma-Aldrich, St. Louis, MO) was added to the homogenate and kept on ice for 30 min. Thereafter, samples were centrifuged at 17,500×*g* for 15 min at 4°C. The supernatants were filtrated using a Millipore Ultrafree MC PLHCC centrifugal filter and analyzed by gas chromatography-mass spectrometry (GC-MS). Then, 1 μl of the sample was injected with a split mode (1:100) into a TRACE GC ULTRA gas chromatograph equipped with a TSQ QUANTUM GC mass spectrometer (Thermo Fisher Scientific, Waltham, MA). A Nukol^™^ fused silica capillary column (0.25 mm ID × 30 m, 0.25 μm film thickness, Supelco, Bellefonte, PA) was used for separation. The column temperature was programmed to 150°C for 2 min, increased to 200°C at a rate of 8°C/min and maintained for 13 min. Helium was used as the carrier gas at a flow rate of 0.7 ml/min. The data were acquired in electron impact ionization mode at 70 eV.

### Induction and evaluation of EAE

EAE was induced in B6 mice by subcutaneous immunization of 50 μg or 100 μg of myelin oligodendrocyte glycoprotein amino acids 35–55 (MOG_35–55_) emulsified in incomplete Freund’s adjuvant containing 250 μg or 500 μg of heat killed *M*. *tuberculosis* H37Ra (Difco, Franklin Lakes, NJ) followed by the intraperitoneal injection of 200 ng of pertussis toxin 48 h later. Clinical signs of EAE were assessed daily as follows: 0, no signs; 1, partial loss of tail tonicity; 2, loss of tail tonicity; 3, partial hind limb paralysis; 4, complete hind limb paralysis; 5, forelimb paralysis of moribund; and 6, death. If mice obrained a score of 5, they were monitored closely every 24 hour. If the mice showed severe weight loss or weakness, they were euthanized. There was no mouse that reached the humane endpoints.

### Induction and assessment of CIA and AIA

To induce CIA, DBA/1J mice were immunized intradermally at the base of the tail with 150 mg of bovine type II collagen (Collagen Research Center, Tokyo, Japan) emulsified with an equal volume of complete Freund’s adjuvant (CFA) containing 250 mg of heat-killed *M*. *tuberculosis* H37Ra (Difco). Three weeks later, mice were given an intradermal injection of 150 mg of type II collagen emulsified in incomplete Freund’s adjuvant. To induce AIA, serum pools were prepared from 8-week-okd arthritic K/BxN mice and B6 mice were given an intraperitoneal injection of 150 μl pooled serum. Clinical assessment of arthritis was scored as follows: 0, no change; 1, significant swelling and redness of 1 digit; 2, mild swelling and erythema of the limb or swelling of >2 digits; 3, marked swelling and erythema of the limb; and 4, maximal swelling and redness of the limb with subsequent ankylosis. The score was expressed as a cumulative value for all paws with a maximum possible score of 16. Humane endpoints for arthritis were: rapid weight loss of >20%, severe paw ulceration, abnormal gait which prevented animals from reaching food or water. There was no mouse that that reached the humane endpoints.

### Histological analysis

For the assessment of EAE, 16 days after the immunization with MOG_35–55_, the spinal cords were sampled and fixed in buffered formalin. Paraffin-embedded spinal cords were stained with hematoxylin and eosin (H&E). For the assessment of arthritis, mice were sacrificed and all 4 paws were fixed in buffered formalin and decalcified. Paraffin-embedded joints were stained with H&E. Joint inflammation was scored as follows: 0, normal joint; 1, mild arthritis (minimal synovitis without cartel/bone erosion); 2, moderate arthritis (synovitis and erosion but joint architecture maintained); and 3, severe arthritis (synovitis, erosion, and loss of joint integrity). The score was expressed as a cumulative value for all paws with a maximum possible score of 12.

### Flow cytometry

Lamina propria lymphocytes (LPL) were isolated according to a standard protocol [16] with minor modifications. Briefly, after the mesentery and fat were removed, excised colons were opened and cut into 0.5-mm pieces. Tissues were washed and incubated in Ca^2+^- and Mg^2+^-free Hank’s buffered saline (HBSS) containing 15 mM HEPES and 5% fetal bovine serum (all from Gibco, Carlsbad, CA) in the presence of 0.5 mM EDTA and 2 mM DTT (GE Healthcare, Fairfield, CT) at 37°C for 40 min with rotation. The supernatant containing epithelial cells and intraepithelial lymphocytes was discarded, and fresh HBSS with EDTA was added and cells were washed. Release of LPL was accomplished by DNase I (15 μg/ml final; Roche, Basel, Switzerland) and collagenase D (1 mg/ml final; Roche) digestion of tissue at 37°C for 50 min with rotation.

The supernatants containing released cells were passed through 70-μm and 40-μm cell strainers, suspended in 40% Percoll (GE Healthcare), overlaid onto 80% Percoll and centrifuged for 30 min at 2,800 rpm at 4°C. Cells at the interface were collected as cLPL. Single cell suspensions of lymph nodes and spleen were obtained by mechanical disruption. For spleen cells, ACK lysis buffer was used to remove red blood cells.

Nonspecific staining was inhibited by incubation with anti-CD16/32 antibody (BD Biosciences, Franklin Lakes, NJ). Cells were then stained with fluorescence-labeled antibodies. Antibodies to CD45 (30-F11), TCRγδ (GL3), TCRβ (H57-597), CD25 (PC61), CD3 (17A2), and CD19 (6D5) were purchased from BioLegend (San Diego, CA). Antibodies to CD4 (RM4-5), CD11b (M1/70), CD11c (HL3), NK1.1 (PK136), and CD25 (PC61) were purchased from BD Biosciences. Dead cells were stained by a LIVE/DEAD Fixable Dead Cell Stain Kit (Invitrogen, Carlsbad, CA). For intracellular FoxP3 staining, an APC Anti-Mouse/Rat FoxP3 staining set (eBioscience, San Diego, CA) was used according to the manufacturer’s protocol. Cells were analyzed by BD FACS Canto II.

### Proliferation and cytokine analysis

For proliferation and cytokine measurement, cells were suspended in RPMI 1640 medium supplemented with 2% autologous serum, 2 mM L-glutamine, 100 U/ml penicillin-streptomycin, and 50 μM 2-mercaptoethanol (2-ME) (Gibco) and stimulated with immobilized anti-CD3 (2C11, 1.0 μg/ml) and anti-CD28 (37.51, 2.0 μg/ml; BD) antibodies for 3 days in 96-well flat-bottom plates. Cells were incubated with [^3^H] thymidine (1 μCi/well) for the final 16 h of culture and incorporation of radioactivity was analyzed by a scintillation counter and expressed as counts per minute. Supernatants were collected and cytokines were measured by using a sandwich enzyme-linked immunosorbent assay (ELISA).

### Recall responses

B6 mice were immunized with MOG_35–55_ in CFA without pertussis toxin. Draining lymph node cells were stimulated with 1–100 μg/ml of MOG_35–55_ for 3 days in RPMI 1640 medium supplemented with 2% autologous serum, 2 mM L-glutamine, 100 U/ml penicillin-streptomycin, and 50 μM 2-ME (Gibco) in 96-well U-bottom plates. Supernatants were collected and cytokines were measured by using a sandwich ELISA.

Differences between data groups were analyzed by one-way or two-way ANOVA and Student’s *t*-test as indicated. *P*-values less than 0.05 were considered significant.

### Real-time RT-PCR analysis

Analysis of the mRNA levels was performed using RT-PCR. C57BL/6 mice were given sodium acetate or sodium propionate or sodium butyrate (Wako) in the drinking water at a final concentration of 200 mM for 2 weeks. Splenic F4/80-positive macrophage were isolated using anti-F4/80 MicroBeads (Miltenyi Biotec, Bergisch Gladbach, Germany) according to the manufacturer's instructions. Total RNA was purified using RNeasy mini kit (Qiagen, Hilden, Germany). First-strand cDNA was synthesized using ReverTra Ace qPCR RT Master Mix (Toyobo, Tokyo, Japan) with 1 μg of total RNA. Real-time RT-PCR with Fast SYBR Green Master Mix (ThermoFisher) was performed using 7500 Fast Real-time PCR System (ThermoFisher) according to the manufacturer’s protocol. mRNA levels were normalized to endogenous glyceraldehyde-3-phosphate dehydrogenase (GAPDH) in each sample. The specific primers used in this study are listed in S1 Table.

## Results

### Fiber-rich diet influences the severity of EAE

**Fig 1:**
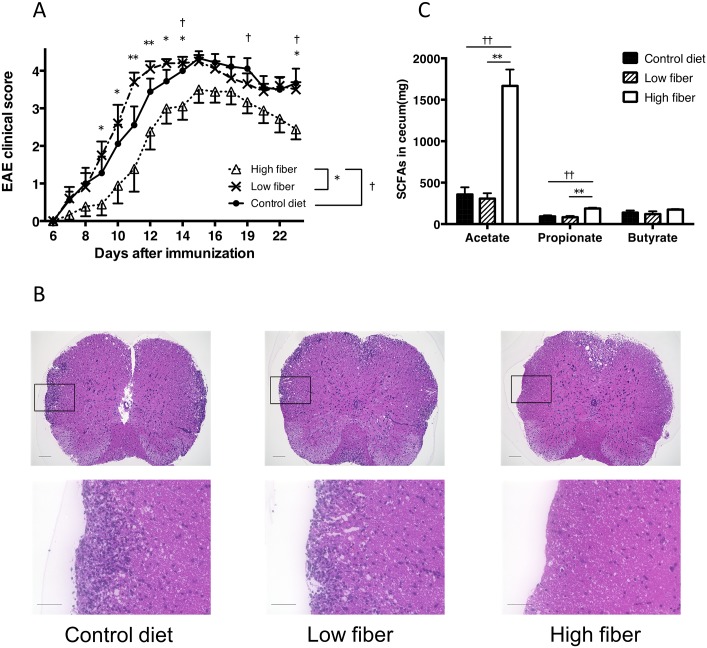
High-fiber diet ameliorates the disease severity of MOG-induced EAE. (**A**) EAE was induced in B6 mice by immunization with MOG_35–55_. Mice were fed a high-fiber, low-fiber or control diet 2 weeks before immunization and throughout the study. *P<0.05, **P<0.005, low-fiber versus high-fiber group. †*P*<0.05, control versus high-fiber group. Data are expressed as the means ± SEM of 9–10 mice per group. **(B)** Histopathological assessment of the CNS in EAE-induced mice. Cellular infiltration of the spinal cord of mice on day 30 is shown. Paraffin-embedded spinal cords were stained with hematoxylin and eosin (H&E). Scale bar = 200 μm (upper panels) or 50 μm (lower panels). **(C)** HPLC quantification of SCFA levels in the cecal contents.

We first examined whether dietary fiber influences EAE by feeding mice with a diet containing high fiber (30% pectin), low fiber (<0.3%) or a control diet (5% cellulose). The disease severity of EAE was reduced in mice fed a high-fiber diet compared with mice fed a regular fiber or low-fiber diet (Fig 1A). Consistent with the clinical scores, histological analysis of the spinal cord 34 days after EAE induction revealed less cellular infiltration and demyelination in mice fed the high-fiber diet compared with mice fed the regular fiber diet (Fig 1B). Because SCFAs such as acetate, propionate, and butyrate are abundant in the metabolites of fermenting fiber, we performed GC-MS analysis of cecal samples from mice receiving the various fiber diets. The levels of acetate, propionate, and butyrate were all increased in the cecal content obtained from mice fed the high-fiber diet compared with mice fed the control or low-fiber diets (Fig 1C).

### SCFAs ameliorate the disease severity of EAE

**Fig 2:**
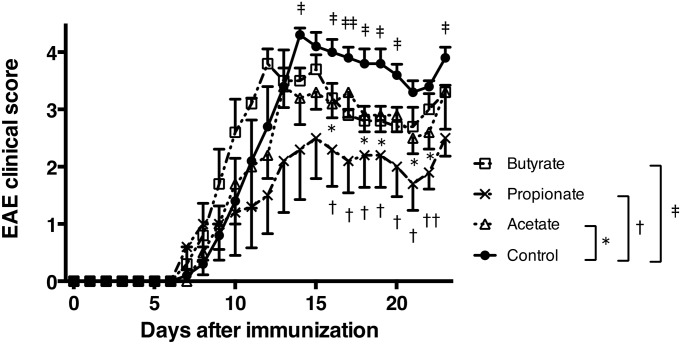
Oral administration of SCFAs ameliorates the disease severity of MOG-induced EAE. EAE was induced in B6 mice by immunization with MOG_35–55_. Mice were orally administrated with SCFAs. *P<0.05, control versus acetate group. †*P*<0.05, control versus propionate group. ‡*P*<0.05, control versus butyrate group. Data are expressed the means ± SEM of 5 mice per group. Representative data from two separate experiments are shown.

**Fig 3:**
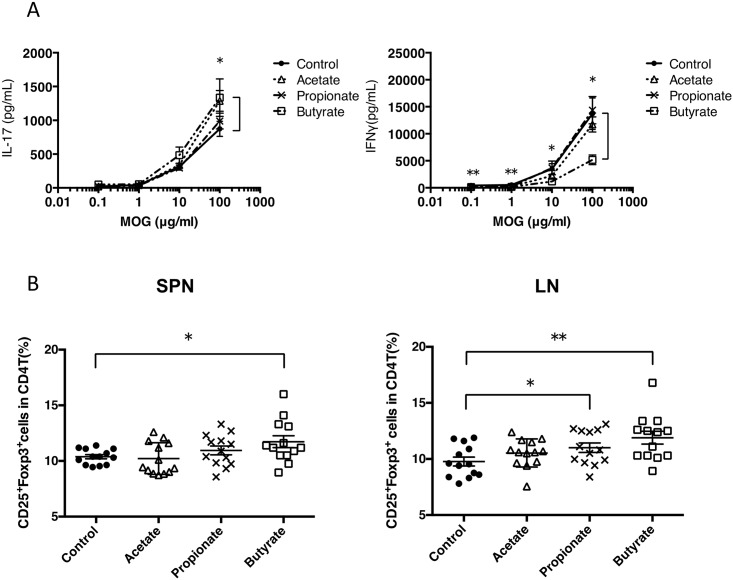
T cell responses in mice orally administrated with SCFAs. (A) B6 mice were administrated with SCFAs and then immunized with MOG_35–55_. Then, 10–11 days after immunization, draining lymph node cells were incubated with MOG_35–55_. Supernatants were collected from the culture and measured for the concentrations of IFN-γ and IL-17 by ELISA. Data represent the mean ± SEM of samples pooled from four similar experiments (n = 13 mice). *P<0.05, ** *P*< 0.01, control versus butyrate group. (B) The frequency of Tregs in spleen (SPN) and draining lymph node (LN) obtained from mice treated with SCFAs were measured using flow cytometry.*P<0.05, ** *P*< 0.005 versus control.

To address the effect of SCFAs on EAE, we treated mice with either acetate, propionate, or butyrate and induced EAE. As in Fig 2, the disease severity was ameliorated in mice treated with acetate or propionate. Butyrate treatment also exhibited a tendency to decrease disease severity (Fig 2). These results indicated that the administration of SCFAs reduced the severity of EAE as well as feeding with a fiber rich diet. We next investigated the effect of SCFAs on the development of MOG-specific Th1 and Th17 cell responses. The production of IFN-γ was inhibited in mice orally administrated with butyrate. In contrast, the production of IL-17 was slightly enhanced in mice treated with SCFAs (Fig 3A). As SCFAs have been reported to increase numbers of Tregs, we examined whether Tregs were altered in these mice. The proportion of FoxP3^+^ Tregs was increased in the spleen and lymph node of mice administrated with SCFAs, particularly butyrate (Fig 3B). These results indicated that the oral administration of SCFAs ameliorated EAE.

### SCFAs reduce the severity of CIA

**Fig 4:**
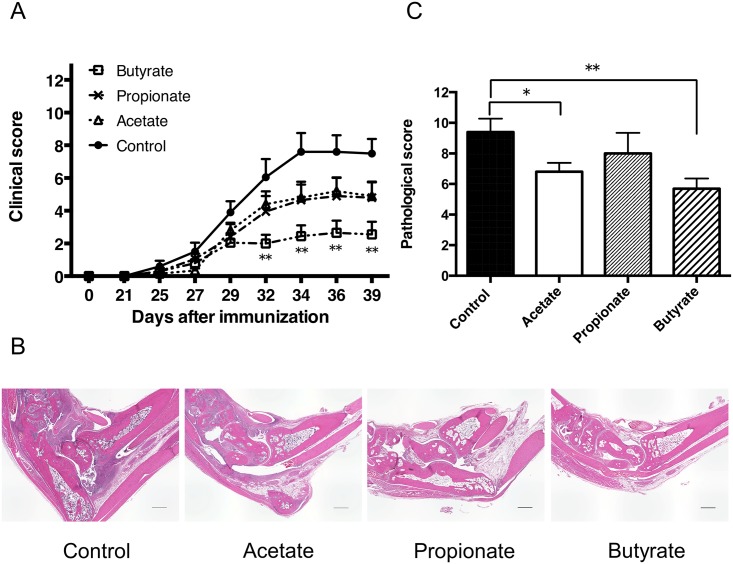
Oral administration of SCFAs ameliorates the disease severity of CIA. (A) Clinical scores of CIA in DBA1/J mice with orally administrated SCFAs 3 weeks before immunization with CII and throughout the study. The data shown are pooled from two similar experiments. Data are expressed as the means ± SEM of 10 mice per group. ** *P*<0.005, control versus butyrate group. (B) Representative histological features of joints in control and SCFAs-treated mice (H&E stained; original magnification ×40). Scale bar = 500 μm. (C) Quantification of histological assessment of joints 46–48 days after induction of CIA. Result shown is the mean + SEM of 5 mice per group. *P<0.05, ** *P*<0.005, versus control group.

Because the administration of SCFA suppressed Th1 responses and increased Treg numbers, we next assessed whether the administration of SCFAs modulated other T cell mediated autoimmune models such as arthritis. We treated mice with either acetate, propionate, or butyrate and induced CIA. As in Fig 4, disease severity was reduced in mice treated with butyrate. Acetate or propionate treatment exhibited a tendency to decrease the disease severity (Fig 4A). Similar to the clinical scores, histological analysis of the joints showed less cellular infiltration, pannus formation, cartilage erosion, and bone destruction in the joints of mice treated with SCFAs. Quantification of the histological severity of arthritis revealed milder joint inflammation in mice treated with SCFAs, particularly butyrate (Fig 4B and 4C). These results suggested that SCFAs contribute to disease amelioration in CIA as well as EAE.

### SCFAs augment inflammation in AIA

**Fig 5:**
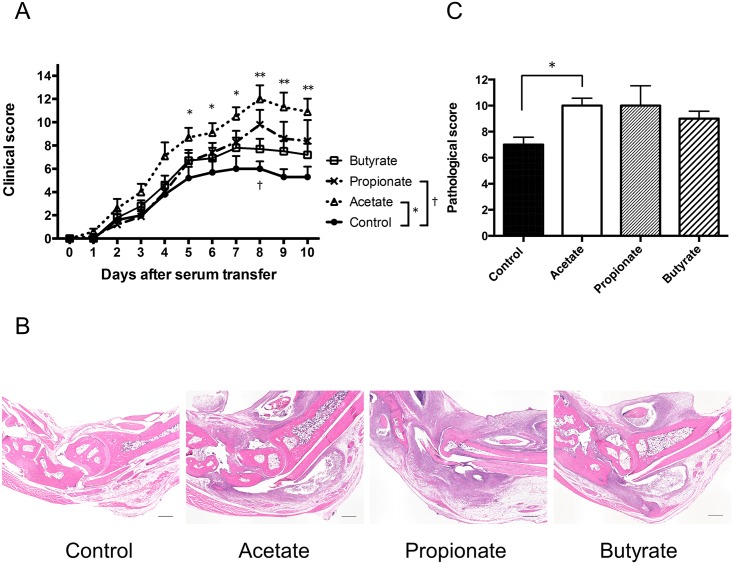
Effect of SCFAs on K/BxN mouse serum–induced arthritis. (A) Clinical scores of K/BxN mouse serum–induced arthritis in B6 mice treated with orally administrated SCFAs 3 weeks before K/BxN mouse serum–induced arthritis induction and throughout the study. *P<0.05, **P<0.005, control versus acetate group. †*P*<0.05, control versus propionate group. Results shown are the mean + SEM of 10 mice per group. The data shown are pooled from two similar experiments. (B) Representative histological features of joints in control and SCFAs-treated mice. (H&E stained; original magnification ×40). Scale bar = 500 μm. (C) Quantification of histological assessment of joints 10 days after induction of K/BxN mouse serum–induced arthritis is shown in A. Results shown are the mean + SEM of 3 mice per group. *P<0.05 control versus acetate group.

**Fig 6:**
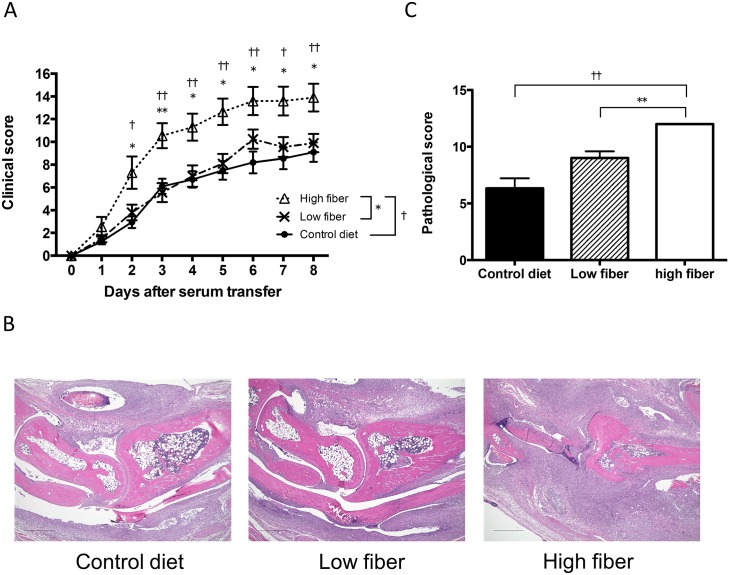
Effect of a high-fiber diet on K/BxN mouse serum–induced arthritis. (A) Clinical scores of K/BxN mouse serum–induced arthritis in B6 mice fed a high-fiber, low-fiber, or control diet 2 weeks before the transfer of serum of K/BxN mouse and throughout the study. *P<0.05, **P<0.005, low-fiber versus high-fiber group. †*P*<0.05, ††*P*<0.005, control diet versus high-fiber group. Results shown are the mean + SEM of 10 mice per group. The data shown are pooled from two similar experiments. (B) Representative histological features of joints in control, low- or high-fiber diet mice (H&E stained; original magnification ×40). Scale bar = 500 μm. (C) Quantification of histological assessment of joints 10 days after induction of K/BxN mouse serum–induced arthritis is shown in A. Results shown are the mean + SEM of 3 mice per group. **P<0.005, low-fiber versus high-fiber group. ††*P*<0.005, control diet versus high-fiber group.

The CIA model requires both adaptive and innate immune responses for disease induction, and the balance of pathogenic Th1 and Th17 cells, and Treg cells, has a role in the inhibition of disease development. In contrast, AIA does not require adaptive immune responses and pathological antibody-induced inflammation in the joints is independent of T cells but dependent on innate immune components. To assess the effect of SCFAs on innate cell-mediated inflammation, we examined the effect of SCFAs on the development of AIA. Unexpectedly, disease development was augmented in mice administrated with SCFAs (Fig 5A). Histological assessment at 10 days after arthritis induction also demonstrated more severe arthritis with cellular infiltration and cartilage and bone destruction in mice administrated with SCFAs (Fig 5B and 5C). We therefore examined whether dietary fiber influenced AIA by feeding mice high-fiber, low-fiber or control diets. Consistent with the administration of SCFAs, disease severity of AIA was more severe by both clinical assessment and histological examination (Fig 6A, 6B and 6C). These findings suggested that SCFAs exacerbate the effector phase of inflammation. To investigate the effect of SCFAs on macrophage, we treated mice with SCFAs for 2 weeks, and measured mRNA expressions of iNOS (M1 marker), Arg-1 (M2 marker), inflammatory cytokines (TNF-α, IL-1β, IL-6), and anti-inflammatory cytokine (IL-10) in splenic macrophages (S1 Fig). The expression of Arg-1 was significantly increased in butyrate treated mice, and the expression of IL-10 showed a tendency of increase in propionate and butyrate treated mice. The treatment of propionate or butyrate, however, induced a significant upregulation of the expression of TNF-α. And there was an increasing trend of the expression of iNOS in propionate and butyrate treated mice. These data indicated that oral treatment of SCFAs altered the phenotype of macrophages, and further studies are needed to reveal the effect of SCFAs to innate immunity including macrophage.

## Discussion

In the present study, we investigated the role of SCFAs in the regulation of inflammation. SCFAs inhibited EAE and CIA associated with an increase in Tregs and a decrease in Th1 cells; however, SCFAs enhanced inflammation in K/BxN serum transfer arthritis.

SCFAs and fiber-rich diets that contribute to the enhancement of SCFA production ameliorated EAE. Previous studies showed that SCFAs inhibited colitis by inducing Tregs in the intestine. Tropette et al. demonstrated that propionate suppressed systemic inflammation such as airway inflammation [14]. More recently, Haghikia et al. reported that the administration of propionate inhibited EAE [15]. Our finding that SCFAs inhibit CIA as well as EAE further supports the idea that SCFAs suppress lymphocyte-mediated systemic inflammation. Consistent with previous studies, we observed an increase in Tregs in mice treated with SCFAs although the difference was not significant in acetate- or propionate-treated mice. The molecular mechanisms of Treg induction by SCFA have already been reported. Furusawa et al. have reported that butyrate promoted the differentiation of Tregs through the inhibition of histone deacetylase and the induction of the upregulation of the histone H3 acetylation of Foxp3 [11]. And Smith et al. revealed that SCFAs induced Foxp3 expression of colonic T cells via activation of free fatty acid receptor 2 (FFAR2) [12]. On the other hand, we observed a slight increase in Th17 cells in mice orally administrated with SCFAs and a decrease in Th1 cells in mice treated with butyrate. The tendency of increased IL-17 upon MOG stimulation was also reported previously consistent with our study [15], although they did not report effects on Th1 cells. Recently, Park et al. reported that the oral administration of SCFAs for a similar duration to our study enhanced the development of Th1 and Th17 cells as well as Tregs, resulting in the augmentation of inflammation in the uterus and kidney [17]. But Gurav et al. reported that dendritic cells exposed to butyrate suppressed conversion of naïve T cells into interferon-γ producing cells [18]. The mechanisms of the reduction of Th1 cells and the induction of Th17 cells by SCFAs require further investigations. The inhibitory effect of SCFAs on CIA was most prominent in butyrate-treated mice, consistent with the inhibition of Th1 cells and an increase in Tregs. Therefore, because the mechanisms that mediate the suppression of EAE following the administration of acetate, propionate, and butyrate are similar, this suggests that other factors contribute to the modulation of disease severity in EAE.

We found that AIA induced by the transfer of K/BxN serum was enhanced by either feeding mice SCFAs or a fiber-rich diet in contrast to CIA. K/BxN mice spontaneously develop a polyarthritis characterized by cellular infiltration, synovial hyperplasia, and bone and cartilage destruction similar to human rheumatoid arthritis [19]. Transfer of K/BxN mouse serum or affinity-purified anti-GPI antibodies induces arthritis in most strains of mice [20, 21]. This adoptive transfer arthritis model requires innate immune cells such as mast cells, neutrophils, and macrophages but not lymphocytes [22–25]. Proinflammatory cytokines such as IL-1 and TNF-α, as well as alternative-pathway complement including C5a and Fc receptor III have been shown to be critical for disease development [22, 26]. On the other hand, the pathogenesis of CIA requires adaptive immune system. It seems that SCFAs may suppress adaptive immunity, especially T cell mediated immunity, but enhance inflammation evoked by innate immune cells. In human, rheumatoid arthritis is a heterogeneous disease, not only in the clinical course and symptoms, but also in the response to treatment, suggesting the heterogeneous and complex pathomechanisms in RA. Thus RA patients may present various response to the SCFA treatment. Several studies of human gut microbiota revealed that SCFA producing bacteria was reduced in the patients of multiple sclerosis or inflammatory bowel disease, but not in the patients of RA [10, 27]. These findings may reflect the complexity in the effect of SCFA to pathology of arthritis.

In our previous study, we reported that IFN-γ suppressed K/BxN serum transfer arthritis [28]. IFN-γ has biphasic functions in several murine arthritis models. For example, IFN-γ exacerbated arthritis at an early stage and suppressed disease severity at a later stage of arthritis [29]. K/BxN serum transfer arthritis is considered to represent the inflammatory process of arthritis. Reduced IFN-γ production caused by the administration of SCFAs may be partly linked to the suppression of K/BxN serum transfer arthritis. GM-CSF also has dual function in autoimmunity and inflammation [30, 31]. In the pathogenesis of EAE, GM-CSF acted as a proinflammatory factor. On the other hand, it has been reported that GM-CSF-treated bone marrow-derived dendritic cells expanded Tregs [32]. In human disease, GM-CSF neutralizing antibody has been reported as a marker of aggressive Crohn’s disease [33]. And GM-CSF was indicated as a treatment for myasthenic crisis in the patients of myasthenia gravis [34]. SCFA has been reported to induce elevating GM-CSF expression in murine monocyte [35]. Therefor GM-CSF may play some role in SCFA-induced Treg expansion. But in arthritis model, GM-CSF has been reported to be indispensable for the pathogenesis of CIA, whereas GM-CSF^-/-^ mice could develop AIA [36]. These findings may explain our conflicting result between CIA and AIA, and the function of GM-CSF in arthritis remains to be elucidated.

In the pathogenesis of arthritis, myeloid cells plays important roles. Macrophages have been reported to be increase in the synovium of RA patients and infiltration of macrophage to synovial membrane correlates with the joint erosion. Recently, it has been reported that butyrate treatment reduced the production of pro-inflammatory cytokines, IL-6 and IL-1β and increased IL-10 production from LPS-stimulated RAW 264.7 cells, which is the murine macrophage cell line [37]. We investigated the effect of SCFAs to macrophages *in vivo*, but we found that oral SCFA-treatment induced upregulation of the expression of both inflammatory and anti-inflammatory genes. Osteoclasts are also known to be a key player in the pathogenesis of arthritis. Butyrate has been reported to suppress the differentiation of osteoclasts [38]. Therefore further studies are required to reveal the effect of SCFAs on osteoclasts *in vivo*.

Recent studies have suggested the association of rheumatoid arthritis and periodontal diseases [39, 40]. *Porphyromonas gingivalis*, one of the major microbes in the pathogenesis of periodontitis, is known to produce SCFAs such as butyrate. Furthermore, butyrate was suspected to be involved in the pathogenesis of periodontitis by inducing reactive oxygen species [41]. Therefore butyrate may enhance inflammation in arthritis under certain conditions. The location of immune activation is an important determinant in immune response. For example, it is reported that administration of type II collagen or MOG into the anterior chamber of the eye induce antigen-specific immune tolerance termed anterior chamber-associated immune deviation (ACAID) [42–46]. Difference of inflammation site may influence the discrepancy of response to SCFAs between CIA and AIA. The further analysis of the mechanisms involved in the augmentation of inflammation remains to be investigated.

## Conclusions

In conclusion, we demonstrated that the oral administration of SCFAs ameliorated the disease severity of lymphocyte-mediated systemic autoimmune inflammatory conditions such as EAE and CIA by reducing Th1 cells and increasing Tregs. In contrast, SCFAs contributed to the exacerbation of antibody-induced inflammation. A further understanding of the effects of microbiota metabolites will lead to the prevention of systemic inflammatory disorders.

## Supporting information

S1 Fig Effect of SCFAs on splenic macrophages. The expression levels of M1 or M2 marker, and inflammatory or anti-inflammatory genes in splenic macrophages obtained from SCFA-treated mice were measured by real-time RT-PCR analysis. Results shown are the mean + SEM of 9 mice per group. The data shown are pooled from three similar experiments. *P<0.05.
(TIF)

S1 Table The sequence of specific primers used in the real-time RT-PCR analysis. (XLSX)
