# Supplementary figures and images for: The dual role of short fatty acid chains in the pathogenesis of autoimmune disease models
Source: PLoS One. 2017 Feb 24;12(2):e0173032. doi: 10.1371/journal.pone.0173032 (PMC5325617; doi:10.1371/journal.pone.0173032)

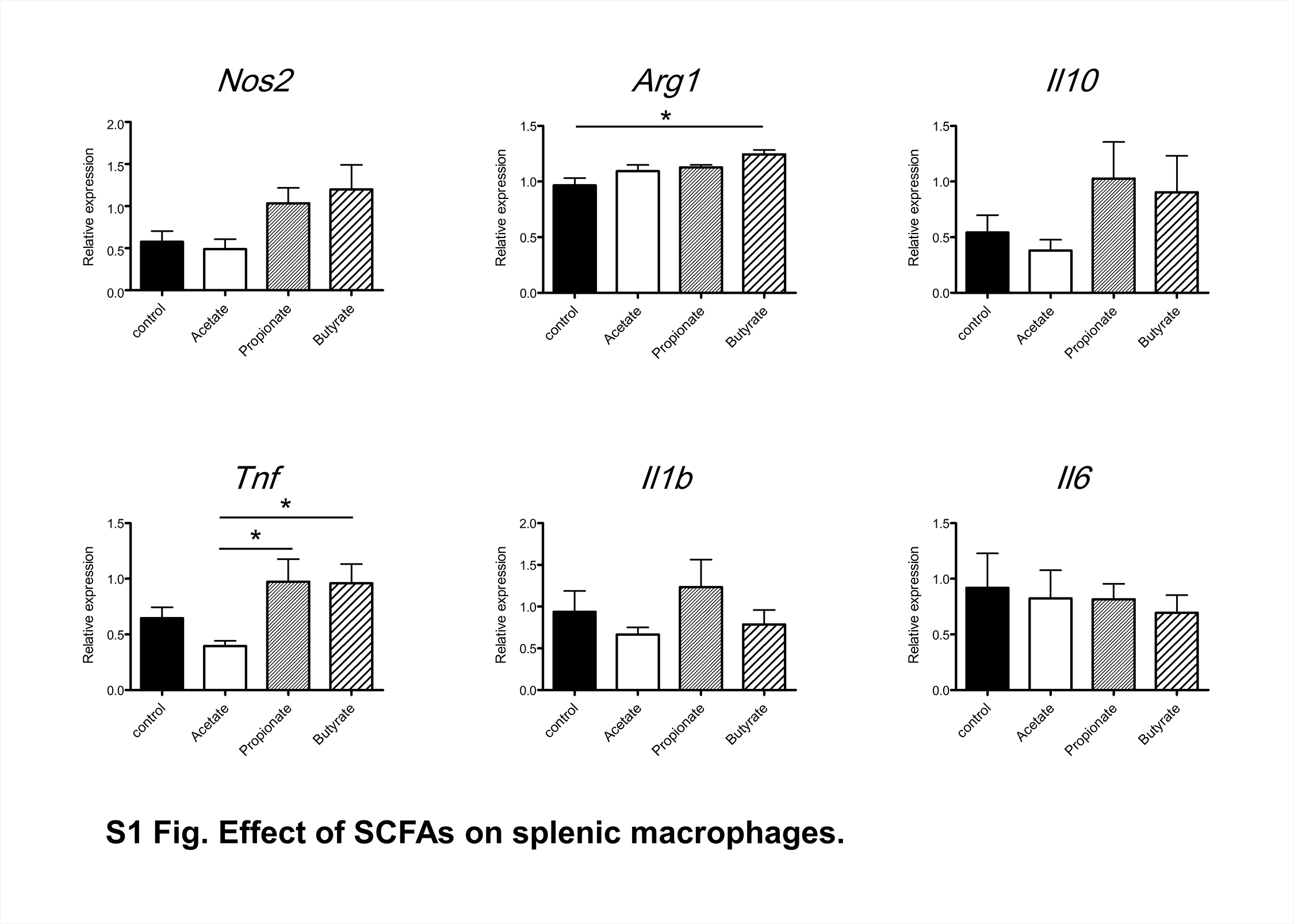

Supplement: S1 Fig — The expression levels of M1 or M2 marker, and inflammatory or anti-inflammatory genes in splenic macrophages obtained from SCFA-treated mice were measured by real-time RT-PCR analysis. Results shown are the mean + SEM of 9 mice per group. The data shown are pooled from three similar experiments. *P<0.05. (TIF) [file pone.0173032.s001.tif]
